# Rational adjustment to interfacial interaction with carbonized polymer dots enabling efficient large-area perovskite light-emitting diodes

**DOI:** 10.1038/s41377-023-01150-1

**Published:** 2023-05-15

**Authors:** Fan Yang, Qingsen Zeng, Wei Dong, Chunyuan Kang, Zexing Qu, Yue Zhao, Haotong Wei, Weitao Zheng, Xiaoyu Zhang, Bai Yang

**Affiliations:** 1 State Key Laboratory of Supramolecular Structure and Materials, College of Chemistry, Jilin University, Changchun, 130012 China; 2 Department of Materials Science, Key Laboratory of Mobile Materials MOE, State Key Laboratory of Automotive Simulation and Control, Jilin University, Changchun, 130012 China; 3 Institute of Theoretical Chemistry and Laboratory of Theoretical & Computational Chemistry, Jilin University, Changchun, 130023 China

**Keywords:** Nanoparticles, Inorganic LEDs

## Abstract

Film uniformity of solution-processed layers is the cornerstone of large-area perovskite light-emitting diodes, which is often determined by the ‘coffee-ring effect’. Here we demonstrate a second factor that cannot be ignored is the solid-liquid interface interaction between substrate and precursor and can be optimized to eliminate rings. A perovskite film with rings can be formed when cations dominate the solid-liquid interface interaction; whereas smooth and homogeneous perovskite emitting layers are generated when anions and anion groups dominate the interaction. This is due to the fact that the type of ions anchored to the substrate can determine how the subsequent film grows. This interfacial interaction is adjusted using carbonized polymer dots, who also orient the perovskite crystals and passivate their buried traps, enabling a 225 mm^2^ large-area perovskite light-emitting diode with a high efficiency of 20.2%.

## Abstract (web summary)

Using carbonized polymer dots, the interfacial properties between perovskites and the hole transporter can be optimized. The anion-dominated interface breeds a dense and homogeneous emitting layer, enabling 20.2% external quantum efficiency for 225 mm^2^ large-area light-emitting device.

## Graphical abstract

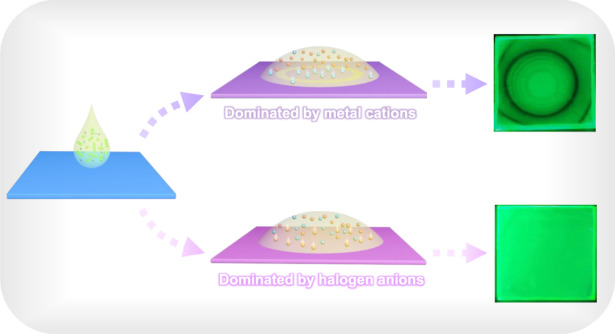

## Introduction

Defect-tolerant lead-halide perovskites are emerging as potential candidates for light-emitting diodes (LEDs) due to their excellent semiconducting properties, low cost, and solution processability^1–5^. With the ability to adjust the bandgaps to cover the whole visible region via dimensional- and compositional-control, as well as narrow emission, perovskites are promising emitters to comply with BT. 2020 (ref. ^6–8^). The family of perovskites includes 3D materials, which are capable of efficient charge carrier transport^9^, and 2D materials, which can convert excitons into photons efficiently^10^. It is expected that a rational structure combining high exciton binding energy and efficient charge carrier transport can be established by adjusting the thickness of [*MX*_6_]^4−^ octahedral layers sandwiched between two organic layers, namely quasi-2D perovskites, a critical path towards efficient electroluminescence (EL)^11–15^. With fundamental advances in understanding device physics and material chemistry, the external quantum efficiency (EQE) of quasi-2D perovskite LEDs (PeLEDs) has risen to over 20%^16–22^. Yet, state-of-the-art devices are generally limited to small areas with only a few square millimeters^16–24^. A noticeable efficiency degradation occurs as the emitting area of the device increases due to optical and morphological inhomogeneities. This problem severely hampers its practical application towards large-area displays.

**Fig. 1:**
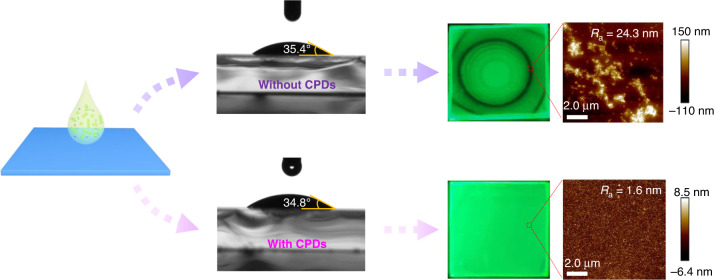
Effects of CPDs on film uniformity of perovskite films. The preparation of quasi-2D perovskite films on different substrates while maintaining the contact angle of the perovskite precursor solution. The significant differences in film morphology suggest that besides the coffee ring effect, other factors determine film quality

The fabrication of large-area perovskite films mainly relies on spin-coating, blade-coating, vacuum deposition, inkjet printing, and spray coating^25^. A smooth, dense, and homogenous perovskite film is essential to high-performance PeLEDs, and the ‘coffee ring effect’ usually influences the fabrication of such films. ‘Coffee ring effect’ occurs when the interior liquids of a droplet migrate to the periphery, carrying solutes deposited at the pinned contact line^26^. Specific methods for suppressing coffee rings include manipulating the substrate temperature or surface energy, solvent engineering, increasing the viscosity of the solution, and adding surfactants^27–30^. Despite effective strategies having been developed to suppress coffee rings^27,31^, the formation of perovskite polycrystalline films, however, involves complex crystallization processes including nucleation, diffusion, and growth rather than simply crystal deposition^32–36^, implying that the ‘Coffee ring effect’ is not the only factor that affects the uniformity of the film, and does not dominate the emergence of rings, indicating existing methods do not effectively inhibit rings from forming. As shown in Fig. 1, when the contact angle of perovskite precursor solution on top of different substrates remains the same, the spin-coated films can either be rough with concentric rings or they can be uniform and compact. This significant contrast suggests that another unknown mechanism determines the film morphology in addition to the coffee ring effect.

In this work, the interaction at the substrate-precursor solid-liquid interface was investigated with the assistance of carbonized-polymer-dots (CPDs), where the substrate is the commercially available poly(3,4-ethylenedioxythiophene):poly(styrenesulfonate) (PEDOT:PSS) hole transport layer (HTL), and the precursor solution is based on a formula for preparing quasi-2D perovskite emitters^37^. There are nitrogen/oxygen-based groups on the surface of CPDs, alongside short polymer chains^38–43^. Metal cations (Pb^2+^ and Cs^+^) dominate the interaction between precursor solution and PEDOT:PSS film, which switches to anions and anion groups (Br^−^, [PbBr_6_]^4−^, [Pb_3_Br_8_]^2−^, etc.) in the presence of CPDs. As a result of different interactions, different types of ions are anchored to the substrate, affecting nucleation, crystallization, and film formation despite the contact angle of the precursor solution being constant. A smooth, dense, and homogenous perovskite film is formed by optimal interactions with CPDs, which also orient perovskite crystals and passivate buried traps. Furthermore, CPD-modified quasi-2D PeLEDs show a high EQE of 24.6% for an active area of 4 mm^2^ and a champion EQE of 20.2% for a large-area of 225 mm^2^, demonstrating that obtaining high-quality large-area perovskite films requires rationally designing the interface interaction between the precursors and underneath substrates. Our initial findings are complementary to what is already known, and the optimization of solid-liquid interface interaction will universally improve the uniformity of films obtained by blade-coating, spraying, spin-coating, etc.

## Results

### Interfacial interaction and film uniformity

A spin-coated quasi-2D perovskite film of BA_2_(CsPbBr_3_)_*n*−1_PbBr_4_ (BA = butylammonium) was deposited on PEDOT:PSS and then thermally annealed (see **Materials and methods Section** for details). The PEDOT:PSS hole transport layer (HTL) was chosen due to its excellent characteristics, including good film forming, high electrical conductivity, and high optical transparency (~ 90% in visible light)^44,45^. There was, however, a series of concentric rings on the prepared perovskite films (Fig. 1, upper row), which severely limited luminous uniformity. As a contrast, the quasi-2D perovskite film deposited on top of the PEDOT:PSS mixed with CPDs (CPD-HTL) is smooth and dense (Fig. 1, bottom row), exhibiting homogeneous photoluminescence (PL).

We fabricated the HTL by spin-coating PEDOT:PSS solution (named p-HTL) or a mixture of the PEDOT:PSS solution with 0.5 mg ml^−1^, 1 mg ml^−1^ and 2 mg ml^−1^ PA-EDA CPDs (named CPD0.5-HTL, CPD1-HTL, CPD2-HTL). CPD1-HTL is selected as representative for subsequent characterization of CPD-HTL. We first tested the time-dependent contact angles of precursor solution atop HTL to identify whether these rings were caused by the ‘coffee ring effect’. The contact line continuously moves outward of both samples during the 4 s period (Figs. 1 and S1), along with the contact angle decrease from 35.2° ± 0.4° to 15.0° ± 0.7°. Unpinning contact lines can reduce the concentration difference between the droplet edge and middle, which in turn inhibits capillary flow and prevent the formation of rings^26,46^, implying that mechanistically it does not support the formation of ‘coffee ring’. Besides, the rings exhibit low photoluminescence (PL), which is also opposite of the ‘coffee ring effect’, where the rings should be bright as a result of grain aggregation. The rings are further examined using atomic force microscopy (AFM). As shown in Fig. 1, inside the dark ring the film is discontinuous as evidenced by the height variation, and it has a poor morphology with a roughness of 24.3 nm. With the introduction of CPDs, on the other hand, produced a uniformly emitting perovskite film that is smooth and compact with a roughness of 1.6 nm. The crystal grains in the ring should be small and dense for solutes driven by the ‘coffee ring effect’ to there can form abundant nucleation center. However, as illustrated in Fig. S2, the morphology of perovskite film on p-HTL is extremely rough in the ring region. The grain size is significantly larger in the ring region than in the non-ring region, which may be due to a reduced number of seed crystals. In the presence of more CPDs, the morphology becomes smoother. According to the above experimental results, we conclude that other factors contribute to the appearance of dark rings rather than the ‘coffee ring effect’.

**Fig. 2:**
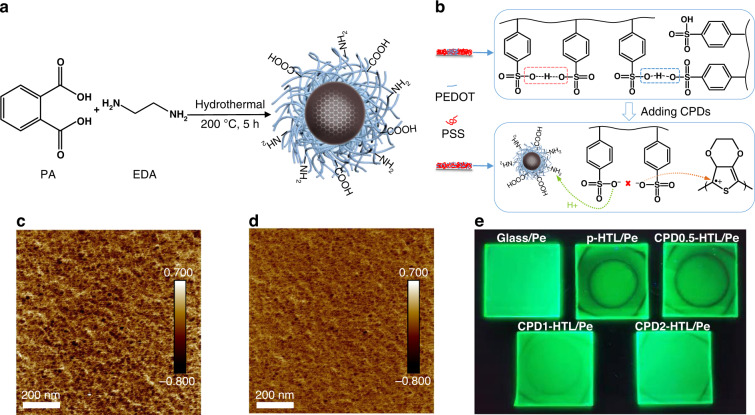
HTLs with CPDs and their effects on perovskite films. **a** A synthesis illustration of the PA-EDA CPDs. **b** A mechanism diagram showing how CPDs inhibit phase separation of PEDOT:PSS. **c**, **d** AFM phase images of (**c**) pristine PEDOT:PSS film and (**d**) PEDOT:PSS film incorporating CPDs. Scale bars on the images are 200 nm. **e** Fluorescence photographs of quasi-2D perovskite films prepared on glass, p-HTL, and CPDx-HTL (x = 0.5, 1, 2) substrates

PEDOT:PSS, the main component of the grain-growth substrate, is rich in sulfonic groups. CPDs, as additive of the grain-growth substrate, contain abundant amino groups. Figure 2a illustrates the one-step hydrothermal synthesis of CPDs from phthalic (PA) and ethylenediamine (EDA). The prepared PA-EDA CPDs are crystalline with a lattice spacing of 0.21 nm, which corresponds to the (100) diffraction facets of graphite^47^, and have an average particle size of 3.17 nm (Fig. S3). XRD analysis shows one broad peak at 21.6° without sharp peaks of small organic molecules (Fig. S4), which indicates the CPDs are well purified and have highly cross-linked polymer skeletons^48^. The Fourier transfer infrared (FTIR) spectrum and X-ray photoelectron spectroscopy (XPS) of CPDs also clearly demonstrate the presence of carboxyl and hydroxyl groups (Figs. S5, S6).

Systematically analysis of interactions between CPDs and PEDOT:PSS, as well as between grain-growth substrate and precursor solution in the presence or absence of CPDs, were conducted to clarify the mechanisms of the emergence and disappearance of concentric rings. For pristine PEDOT:PSS, phase separation occurs during spinning because the hydrogen bonds based on -SO_3_H groups provide cohesive strength between PSS polymer chains^49^. This kind of hydrogen bond reduces with the appearance of CPDs, due to the amino groups in PA-EDA CPDs present a mechanism for binding hydrophilic hydrogen ions in -SO_3_H groups (Fig. 2b). Fig. S7 confirms the strong interaction between CPDs and PEDOT:PSS, by comparing photos of their mixtures with different CPDs contents and types, demonstrating that amino groups dominant this interaction. Fig. S8 shows that the acidity of PEDOT:PSS solution decreased after introducing PA-EDA CPDs, which indicates that hydrogen ions dissociated from -SO_3_H groups are consumed by protonating amino groups. Following interaction with PEDOT:PSS, CPDs show a higher N 1 *s* XPS peak, shifting from 400 eV to 402 eV, revealing the conversion from amino to protonated amino groups (Figs. S6d, S9). S 2*p* XPS spectra and the atomic area ratio of -SO_3_H group to $- {{{\mathrm{SO}}}}_3^ -$ group (${A}_{{\hbox{-}{{{\mathrm{SO}}}}}_{3}{\mathrm{H}}}/A_{\hbox{-}{{{\mathrm{SO}}}}_{3}^{-}}$) further prove the deprotonation of -SO_3_H groups in CPDs-incorporated HTL (Fig. S10 and Table S1). The protonated amino groups replace -SO_3_H groups to bind with $- {{{\mathrm{SO}}}}_3^ -$ groups through electrostatic interaction, thus avoiding aggregation of PSS chains. AFM phase images were taken to compare the microstructure of pristine and PA-EDA CPDs doped PEDOT:PSS films (Fig. 2c, d). Bright and dark features in phase images are interpreted as stiff PEDOT-rich and soft PSS-rich regions, respectively^50^. Figure 2c, d suggest that PEDOT and PSS have been blended homogeneously with the appearance of CPDs. The electrical properties of CPDs-incorporated PEDOT:PSS thin films were investigated by conducting electrical conductivity measurements using linear sweep voltammetry. With the introduction of CPDs, PEDOT:PSS films became less conductive, as demonstrated in Fig. S11. This can be attributed to a reduced phase separation between high-conductive PEDOT regions and insulated PSS regions (Fig. 2c, d). The element distribution in Fig. S12 and AFM morphology in Fig. S13 indicate that PA-EDA CPDs are uniformly doped in PEDOT:PSS films.

As a result of its interaction with perovskite intermediate phases, the grain-growth substrate plays an important role in fostering the nucleation and growth of perovskite crystals. This suggests that the existence of these rings may be attributable to the circular distribution of intermediate phases driven by solid-liquid interfacial interactions. For PEDOT:PSS, their sulfonic group can provide three oxygen atoms to coordinate with Pb^2+^, making it a promising candidate for strong interactions with perovskite intermediate phase. The characteristic S = O symmetric stretching vibration peak for pristine PEDOT:PSS is shown in Fig. S14a, which shifts from 1038 cm^−1^ to a lower wavenumber of 1033 cm^−1^ when interacting with Cs^+^, then to a wavenumber of 1031 cm^−1^ when interacting with Pb^2+^. The peak spreads clearly in terms of S = O asymmetric stretching vibrations, as strong interactions between $- {{{\mathrm{SO}}}}_3^ -$ and Pb^2+^ break the symmetry of the $- {{{\mathrm{SO}}}}_3^ -$ groups. The PEDOT-related peaks do not exhibit any shift, demonstrating a weak interaction owing to the large steric hindrance of coordination atoms. According to Fig. S15, the Pb 4 *f* XPS signal of perovskites has significantly lower binding energy on PEDOT:PSS compared with ITO glass due to electron donation from O atoms, which is in agreement with the FTIR results. A strong interaction between the sulfonic groups in PSS and Pb^2+^ in intermediate phase has been confirmed by both the FTIR and XPS data.

The interaction between PEDOT:PSS and precursor solution takes a radical turn with the appearance of CPDs. Spectra of ^1^H nuclear magnetic resonance (NMR) in Fig. S16 are first used to examine the possible interaction between CPDs and perovskite precursors. Following simple acidification of CPDs (fully mixing CPDs with trace sulfuric acid, named CPDs–H), the amino-related hydrogen signal at 2.738 ppm disappears, a broad characteristic peak at 8.790 ppm corresponding to protonated amino appears instead. For CPDs-H:CsBr or CPDs-H:PbBr_2_ mixtures, the protonated amino resonance peaks shift to higher fields, implying interactions between the Br^−^ and –NH_3_^+^. FTIR spectra in Fig. S14b further support the interaction between Br^−^ and –NH_3_^+^, as NH_3_^+^ peak variation is consistent in both cases with CsBr and PbBr_2_ added. As a means of better understanding the interaction between grain-growth substrate and precursor solutions in the presence or absence of CPDs, density functional theory (DFT) calculations were conducted (Table 1). According to the calculations, the $- {{{\mathrm{SO}}}}_3^ -$ groups have high binding energy with metal cations (−2.544 eV with Pb^2+^, −0.379 eV with Cs^+^), but their electrostatic repulsion prevents them from bonding with anions or anion groups. For CPDs with abundant protonated amino groups, they bind to anions or anion groups more strongly (−0.441 eV with Br^−^, −1.035 eV with [PbBr_6_]^4−^). Furthermore, the Pb 4 *f*, Cs 3*d* and Br 3*d* XPS signals of quasi-2D perovskites have significantly higher binding energies on CPD-PEDOT:PSS than on PEDOT:PSS substrate according to Fig. S15, which indicates a weakened electron donation from the substrate to cations as well as an enhanced electron-withdrawing effect to Br^−^ with the appearance of CPDs. Theoretical calculations are in line with the experimental results, indicating the incorporation of CPDs at the interface can weaken the interaction between substrate and cations, and enhance the interaction between substrate and anions. By regulating the content of CPDs, the absorbed ions types can be adjusted accordingly. Due to the higher concentrations of halogen ions over Pb^2+^ or Cs^+^, they are not sensitive to variation in concentration caused by interface adsorption, so that uniform film formation is likely to occur (Fig. S17). Metal cations anchored at the interface can evolve into defect centers, which weaken the fluorescence. Alternatively, pulling more anions in the presence of CPDs inhibits the appearance of defects. As a consequence, the concentric rings on quasi-2D perovskite films gradually decrease, fade and even disappear as the contents of CPDs in HTL increase (as shown in Fig. 2e). As demonstrated above, PA-EDA CPDs are effective in controlling substrate-precursor interface interactions and enhancing uniformity of perovskite films by adjusting anchored ion types.

**Table 1:** DFT calculation of the binding energy *E*_b_ (eV) of $- {{{\mathbf{SO}}}}_3^ -$ and –NH_3_^+^ with perovskite ions

	$- {{{\mathbf{SO}}}}_3^ -$	–NH_3_^+^
Pb^2+^	−2.544	—
Cs^+^	−0.379	−0.026
Br^−^	—	−0.441
[PbBr_6_]^4−^	—	−1.035

### Perovskite orientation and phase distribution

**Fig. 3:**
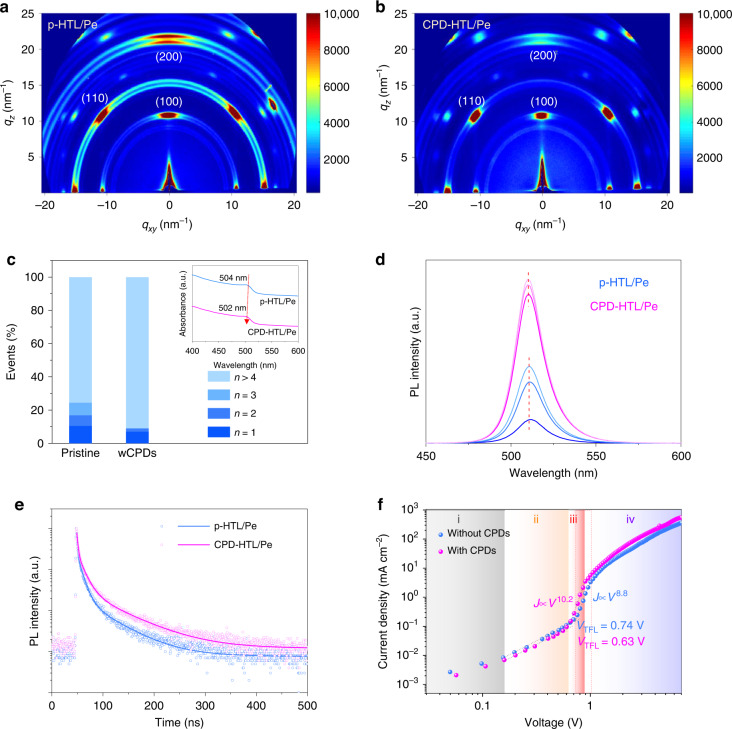
Structure and characteristics of quasi-2D perovskite films. **a**, **b** 2D GIWAXS patterns of (**a**) p-HTL/Pe and (**b**) CPD-HTL/Pe films. **c** The relative contents of different *n* phases of p-HTL/Pe and CPD-HTL/Pe films. The insert shows the bandgap change of large-*n* phase with the appearance of CPDs. **d** Center- and edge-region PL spectra for p-HTL/Pe (blue) and CPD-HTL/Pe (pink). **e** TRPL decay of p-HTL/Pe and CPD-HTL/Pe films. **f** Current density-voltage (*J*-*V*) curves of hole-only devices measured in the dark with the structure of ITO/p-HTL or CPD-HTL (~25 nm)/quasi-2D perovskite (~25 nm)/MoO_*x*_ (~1 nm)/ Ag (~100 nm). There are four different regions in the *J*-*V* curves: (i) Ohmic region (*J* **∝** *V*), conductivity is determined by background charge carriers, (ii) trap SCLC region (*J* **∝** *V*^2^), (iii) trap-filled region (*J* **∝** *V*^m^, m> 2), and (iv) SCLC region in the absence of traps (*J* **∝** *V*^2^)

According to the interfacial interaction mechanism revealed above, details regarding the effects of CPDs on perovskite films were studied. As properties are determined by structure, our first step was to investigate the crystal structure and orientation of perovskite films in order to fully understand their properties. Grazing-incidence wide-angle X-ray scattering (GIWAXS) measurement was used to probe the structure changes of perovskite crystals. It can be seen in Fig. 3a, b that three main planes (100), (110), and (200) faces can be recognized in quasi-2D perovskite films prepared on pristine HTL (named p-HTL/Pe) and PA-EDA CPDs mixed HTL (named CPD-HTL/Pe)^51^. As compared to p-HTL/Pe, CPD-HTL/Pe exhibits a preferential orientation with weaker anisotropic diffraction rings^52^. Also, the diffraction intensity of the (100) and (200) planes is decreased in the out-of-plane direction (*q*_*z*_), but increased in the in-plane direction (*q*_*r*_), suggesting less parallel and more vertical orientations^53,54^. The difference in interaction between grain-growth substrates and precursor solutions causes this change. Due to the strong interaction between p-HTL substrate and metal ions, nucleation distribution and subsequent crystallization are disrupted, leading to a random orientation. CPD-HTL/Pe, on the other hand, dramatically changed nucleus types and reconstructed film structure by strengthening anion interactions and weakening cation interactions. As ordered crystal arrangements improve carrier transport, we expect LED devices based on CPD-HTL/Pe films to have a higher electroluminescence (EL) efficiency.

Energy acceptor-to-donor ratio, or phase distribution, is another key determinant of EL performance for quasi-2D perovskites. An increased ratio of large-*n* phase with narrow bandgap quantum wells can smooth the energy transfer pathway, leading to greater efficiency in emission. We proceeded to investigate the phase distribution of quasi-2D perovskites by estimating the relative content of different *n* phases from absorption spectra, whereby four absorption peaks at wavelengths around 411 nm, 433 nm, 463 nm, and 500 nm are identified as *n* = 1, 2, 3, and >4 phases, respectively (Figs. 3c, S18)^17,55,56^. As compared to p-HTL/Pe, CPD-HTL/Pe exhibits a narrower phase distribution with negligible absorption from small-*n* phases and concentrated absorption of large-*n* phases. It was also observed that the bandgap of the large-*n* phase increased from 2.46 eV to 2.47 eV, which is due to the efficient defect passivation, as more will be discussed later. To evaluate the energy transfer and carrier dynamics in CPD-HTL/Pe, we performed ultrafast transient absorption (TA) spectroscopy. TA spectra of p-HTL/Pe and CPD-HTL/Pe at variable timescales are shown in Fig. S19a, b, in which typical ground-state bleaching (GSB) peaks can be observed. The reference peaks around 406, 430, 460, and 508 nm can be assigned to the quasi-2D phase corresponding to *n* = 1, 2, 3, and ∞ , respectively. It is notable that the *n* = 1 phase almost disappeared in CPD-HTL/Pe, which confirms the suppressed phase disproportionation caused by CPD regulation. The *n* =∞ phase in CPD-HTL/Pe shows a blueshift compared with p-HTL/Pe film, which is consistent with the steady-state absorption spectra. As the delay time prolongs, small-*n* GSB peaks diminish accompanied with a gradual increase of the high-*n* peak, implying the energy transfer from small-*n* phases to high-*n* phases. Fig. S19c, d show the kinetic profiles extracted from the *n* =∞ phase for both perovskite films, which are fitted using multi-exponential functions to investigate carrier dynamics. The profiles rise and decay with time scales, containing a rapid time (*τ*_1_) corresponding to the establishment of bleaching peaks and a decay with fast (*τ*_2_) and slow moment (*τ*_3_) relating to complicated decay processes^53,57^. The fitted parameters are summarized in Table S2. Compared with p-HTL/Pe, CPD-HTL/Pe has a shorter *τ*_1_ of 0.882 ps, indicating that suppression of small-*n* phases accelerates the energy transfer process. According to the above results, the regulation of interfacial interaction by CPDs suppresses phase disproportionation and enables regular orientation of perovskites, which is conducive to efficient luminescence and charge transport.

### Buried-defect passivation

We analyzed the luminescence properties by conducting photoluminescence quantum yield (PLQY) analysis. The CPD-HTL/Pe film shows a much higher PLQY of ~85% than that of ~55% for the p-HTL/Pe film. The steady-state PL spectra and time-resolved photoluminescence (TRPL) spectroscopy analysis also confirmed higher PL intensity and longer PL lifetimes in the CPD-HTL/Pe film compared to the p-HTL/Pe film (Fig. 3d, e). Enhanced energy transfer between small-*n* and large-*n* phases, in combination with properly passivated trap states (undercoordinated Pb atoms and halide vacancies that act as non-radiative recombination centers in the perovskite emitter), results in dramatic improvements in radiative recombination. Besides, the PL intensity and peak position in CPD-HTL/Pe film are almost constant across different regions, agreeing with its uniform phase distribution, while PL in p-HTL/Pe film is weaker in the edge area, with discrepant peak locations due to uneven film morphology. Using emitting layers with uniform PL intensity and constant PL peak position, such as CPD-HTL/Pe films, can be advantageous for large-area EL devices.

To further confirm the passivation effect of the CPD-HTL/Pe film, we performed trap-density analysis by fabricating a hole-only device and analyzing the transport characteristics. The space charge limited current (SCLC) method is used to quantify trap density (*N*_t_). A hole-only device with CPD-HTL/Pe film exhibits lower current density at Ohmic and trap SCLC regions than the one with p-HTL/Pe, indicating effective leakage current suppression. The thickness of perovskite layer on different HTLs were measured to around 25 nm (Fig. S20). The *N*_t_ was calculated to reduce from 6.29 × 10^17 ^cm^−3^ to 5.35 × 10^17 ^cm^−3^, according to the formula $N_{{{\mathrm{t}}}} = 2\varepsilon _r\varepsilon _0V_{{{{\mathrm{TFL}}}}}/eL^2$, where *ε*_*r*_ is the relative permittivity of perovskite (4.8), *ε*_0_ is the vacuum permittivity, *V*_TFL_ is trap-filled limit voltage (observed to decrease from 0.74 V to 0.63 V), *e* is the elementary charge, and *L* is the thickness of perovskite layer^58,59^. Further, the steep slope of *J-V* curves for the device with CPD-HTL/Pe in the trap-filled limited region is steeper, indicating that vertical orientation effectively enhances hole transport, which is crucial for EL devices.

### Light-emitting diode performance

**Fig. 4:**
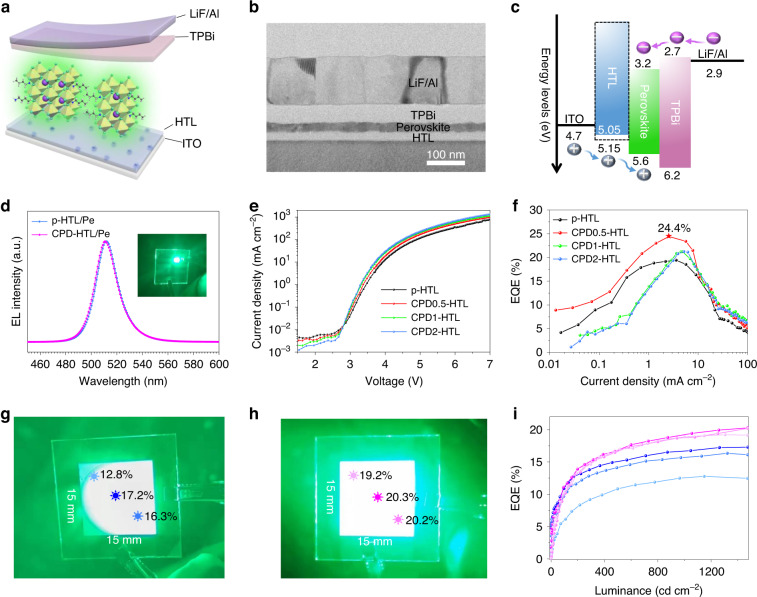
Device structure and performance of Quasi-2D PeLEDs. **a** Schematic illustration of the multilayered PeLED. **b** Cross-section TEM image of the quasi-2D PeLED. **c** Schematics of energy diagram of the multilayered PeLED. HTL and CPD-HTL energy levels are obtained from UPS measurement, while other energy levels are derived from the literature^53,68^. **d** EL spectra of the PeLEDs with and without CPDs, with a photograph showing a working device. **e** Current density versus voltage. **f** EQE versus current density under optimized conditions based on control devices. **g**, **h** Photographs of 225 mm^2^ PeLEDs (**g**) without and (**h**) with CPDs (CPD2-HTL). **i** EQE versus luminance curves of the marked points in (**g**) (blue line) and (**h**) (pink line)

With increased PL efficiency, decreased trap density, and fast charge transport, we fabricated LEDs using CPD-HTL/Pe films. The influence of PA-EDA CPDs on device performance was investigated by characterization of multi-layered LEDs fabricated using the structure of ITO/CPD-HTL or p-HTL/quasi-2D perovskite/TPBi/LiF/Al (Fig. 4a). According to cross-section TEM image (Fig. 4b), the thickness of HTL, perovskite emitting layer, TPBi, and LiF/Al are around 25, 25, 45, and 120 nm, respectively. Figure 4c presents the device energy level diagram of this multi-layered structure. Ultraviolet photoelectron spectroscopy (UPS) shows a deeper work function (WF) for CPD-HTL than p-HTL (Fig. S21), which is conducive to reducing the hole injection barrier into perovskites. As shown in Fig. 4d, the EL peak exhibits a slight shift from 512 nm to 510 nm with the use of CPDs, which is consistent with defect passivation. The Commission Internationale de L’Eclairage (CIE) chromaticity coordinate is (0.08, 0.72). In particular, the EL spectra of devices with CPDs (Fig. S22) exhibit high stability against applied bias voltage, indicating that the transport of electrons and holes is balanced, and perovskites serve as primary emitting centers.

The *J-V* curves of PeLEDs with different HTL are presented in Fig. 4e. PeLEDs with CPDs have a turn-on voltage of 2.68 V, which is lower than that of the device without CPDs (2.82 V). Devices with CPDs currently have a much lower density before turning on than devices without, which is due to the improved morphology of the perovskite films. The current density of LEDs with CPDs increases faster than that of LEDs without CPDs when they are turned on, because CPDs boost carrier injection into the perovskite emission layer. With increasing voltage, the brightness of devices with CPDs increases more dramatically (Fig. S23a). A number of factors contribute to the optimized device performance, such as the ordered crystal orientation, reduced defects, and matching of the energy levels with the regulation of CPDs. Without CPDs, the devices achieve a maximum current efficiency of 58.6 cd A^−1^ (Fig. S23b) and an EQE of 19.4% (Fig. 4f), which are above average among quasi-2D PeLEDs^60^. With CPDs, the devices show a high current efficiency of 73.7 cd A^−1^, and its maximum EQE reaches 24.4%, which is among the highest values in green quasi-2D LEDs. The maximum efficiencies of devices with more CPDs are obtained by rebuilding the balance between holes and electrons within the perovskite emitters due to the influence of CPDs on the conductivity of HTL (Fig. S11). With proper regulation of ETL thickness, devices with CPD1-HTL reached an EQE of 24.6%, while devices with CPD2-HTL reached an EQE of 24.3% (Fig. S24). The operational stability of quasi-2D PeLEDs was investigated in a glovebox filled with nitrogen without encapsulation. Fig. S25 shows that the CPDs-modified device has a *T*_50_ operation lifetime (the time for the luminance to decrease to half of the initial luminance) of 51 min, which is much longer than the 9 min for the device without CPDs.

With CPD-HTL/Pe films of excellent uniformity, it is possible to fabricate large-area PeLEDs. The photographs of the devices with emitting areas of 225 mm^2^ provided in Fig. 4g, h represent devices without and with CPDs. The presence of concentric rings on pristine perovskite films seriously degrade the device performance and emission uniformity (Fig. 4g). By contrast, LED with CPDs exhibits bright, uniform EL with an EQE that is around 20% regardless of the device’s center or edge region (Fig. 4h, i). As a means of reflecting the uniformity of large-area films, the efficiency distribution of small-area devices are compared by Fig. S26 and Fig. S27. While the EQE distribution is narrow for devices with CPD-HTLs, the EQE distribution for PeLEDs without CPDs varies from 5% to 17%, indicating poor unity. EQE of 20.2% was achieved from these CPD modified large-area LEDs, a record efficiency in large-area quasi-2D PeLEDs for all colors, to the best of our knowledge.

## Discussion

We demonstrated how to control the interfacial interactions at the substrate-precursor solid-liquid interface by introducing CPDs into PEDOT:PSS HTL, towards uniform quasi-2D perovskite films with a low trap density. In the absence of CPDs, interactions at the solid-liquid interface are dominated by cations, causing perovskite films with rings. In the presence of CPDs, smooth and homogeneous perovskite emitting layers are generated because of anions and anion groups dominating the interaction. The optimized interfacial interactions enable oriented crystal growth, which allows for efficient charge transport. The rich surface chemistry of CPDs contributes to defect passivation, which lowers trap density and increases PLQY. As a result, we achieved a record efficiency (EQE of 20.2%) for large-area quasi-2D PeLEDs (225 mm^2^) with simultaneous high brightness and uniform EL. This work offers original thinking for fabricating uniform perovskite films of large areas, and a promising approach for fabricating high-performance large-area PeLEDs based on quasi-2D perovskites.

## Materials and methods

### Materials

All chemicals were used without further purification. Phthalic (PA, 99.5%) and citric acid (CA, 99.5%) were purchased from Aladdin. Ethylenediamine (EDA, 99%) and cesium bromide (CsBr, 99.999%) were purchased from Alfa Aesar. Poly(ethylene oxide) (PEO, average Mv 100,000), lithium fluoride (LiF, 99.98%), n-butylammonium bromide (BABr, 98%), and dimethyl sulfoxide (DMSO, ≥ 99.9%) were purchased from Sigma Aldrich. Lead bromide (PbBr_2_ 98%) was purchased from TCI. Poly(3,4-ethylenedioxythiophene):poly(styrenesulfonate) (PEDOT:PSS) (4083) and 1,3,5-Tris(1-phenyl-1H-benzimidazol-2-yl)benzene (TPBi, 99.5%) were purchased from Xi’an Polymer Light Technology Corp.

### Synthesis of CPDs

The PA-EDA CPDs were synthesized by hydrothermal treatment of phthalic (PA) and ethylenediamine (EDA). Typical synthesis involves dissolving PA (0.005 mol, 0.83 g) and EDA (0.01 mol, 0.67 mL) in 40 mL deionized water with a stoichiometric ratio of 1:2. Following 15 min of sonication, the transparent solution was transferred to a poly(tetrafluoroethylene) (Teflon)-lined autoclave and heated at 200 °C for 5 h. Then the as-prepared production was naturally cooled down to room temperature and filtered through a 0.45 μm polyethersulfone filter membrane to remove the large particles. Consequently, a clear yellowish solution was obtained. Afterward, CPDs solution was dialyzed against deionized water for 24 h to remove any excess precursors or byproducts, using a 500–1000 molecular weight cut-off. Freeze-drying was used to remove excess water and obtain a flavescent power. A similar method was used for amino-free CPDs, with CA serving as the sole carbon source.

### Preparation of the mixed solution of PEDOT:PSS and CPDs

Various amounts of PA-EDA CPDs (0.5 mg, 1 mg, and 2 mg) were added to PEDOT:PSS solution (1 mL) and vigorously stirred for 20 min. We filtered this mixture through a 0.45 μm polyethersulfone filter membrane before using it.

### Preparation of quasi-2D perovskite films

Quasi-2D perovskite films were prepared according to references^16^. Firstly, the perovskite precursor solution was prepared by dissolving CsBr, PbBr_2_, BABr with a ratio of 1:1.2:0.4, and PEO (3 mg) in anhydrous DMSO (1.5 mL). The mixture was stirred overnight at 60 °C, cooled to room temperature, and filtered through a 0.45 μm Nylon 6 filter membrane. As a final step, the precursor solution was spun-coated at 4000 rpm for 40 s in a nitrogen-filled glovebox and then annealed at 70 °C for five minutes on a hot plate.

### Fabrication of PeLEDs

A structure of glass/ITO/p-HTL or CPD-HTL/quasi-2D perovskite/TPBi/LiF/Al was used to fabricate the PeLEDs. Ultrasonic cleaning of ITO-patterned glass substrates was performed using detergent, deionized water, trichloromethane, acetone, isopropyl alcohol, and ethanol in sequence. Prior to layer deposition, the ITO-patterned glass substrates were dried with nitrogen and treated with UV plasma for 15 min. Filtered PEDOT:PSS:CPDs solution was spin-coated onto ITO glass substrates at 4000 rpm for 40 s, followed by annealing at 140 °C for 15 min. The substrates were transferred into a nitrogen-filled glovebox and spin-coated with perovskite precursor solution. Under a high-vacuum of less than 10^−4 ^Pa, the fabrication process was completed by depositing TPBi (45 nm), LiF (1 nm), and Al (120 nm) in a vacuum evaporation system. The device’s effective area was 4 mm^2^ as defined by the overlapping area of ITO and Al electrodes. In the same way as above mentioned, large-area devices with an active area of 225 nm^2^ were also fabricated.

### Characterization techniques

A Dataphysics OCA20 was used in the air to measure the contact angles of perovskite precursor solution on substrates. A JEM-2100F microscope operating at 200 kV with super-thin carbon films was used for TEM and high-resolution TEM characterizations of CPDs. Focused ion beam technique was combined with TEM to obtain cross-section TEM images of a PeLED, which was deposited on a Si substrate and treated by Helios 5 ux. AFM images were obtained by a Bruker Nanoscope IIIa scanning probe microscope from Digital Instruments in a tapping mode under ambient conditions. Fluorescence spectra were obtained by Shimadzu RF-5301 PC spectrometer. UV − vis absorption spectra were measured using a Shimadzu UV-3101PC spectrophotometer. XPS data was collected by ESCALAB250 from THERMO FISHER SCIENTIFIC. FT-IR spectra were measured on a VERTEX 80 V FT-IR spectrophotometer from Bruker. ^1^H NMR spectra were recorded on a Zhongke-Niujin AS400 instruments at room temperature utilizing DMSO-d6 as solvent. FiveEasy Plus pH detector with a InLab Viscous ProISM electrode from Mettler Toledo was used to monitor the pH of solutions. The electrode has a temperature compensation function and was calibrated with standard solutions before use. Each pH value in this article has been measured three times order to ensure the reliability of data. EDS and mapping measurements for elemental analysis were performed with HITACHI FEI energy disperse spectroscopy. GIWAXS was measured at Synchrotron Radiation Research Center (Taiwan, China) and was provided technical support by “Ceshigo Research Service”. All samples for GIWAXS were radiated at 12 keV X-ray with an incident angle of 0.1°. TRPL measurements were performed on a time-correlated single-photon counting system under right-angle sample geometry using FLS920 fluorescence lifetime spectrometer (Edinburgh Instruments). A 360 nm picosecond diode laser was used to excite the samples. The PL decay time was obtained at the optimal emission wavelength for the quasi-2D perovskite. PLQYs were collected by an integrating sphere with a QE Pro spectrometer. The perovskite films were excited by a 405 nm laser in a nitrogen-filled glovebox. R3000 high resolution ultraviolet photoemission spectrometer from PREVAC was used to calculate the energy band. The measurement was performed under ultrahigh vacuum with monochromatic UV light source He I (21.2 eV). The work function (WF) was calculated by the following formula: WF = *hν* - *E*_cutoff_, where *hν* = 21.22 eV, *E*_cutoff_ is the steep edge position in UPS spectrum. The EL spectra and CIE chromaticity coordinate characterization of LEDs were measured using a Keithley 2400 source meter and a SpectraScan® Spectroradiometer PR-735. Current density-voltage characteristics of hole-only devices and PeLED devices were tested by a Keithley 2612 A system source meter in a nitrogen-filled glovebox without encapsulation. The light output was measured by a calibrated Newport 1936-R power meter with a 918D-SL-0D3R silicon photodetector positioned at a fixed distance and directed toward the ITO glass side of the LED device in the darkroom.

### DFT simulations

All calculations were performed using density functional theory (DFT) within Gaussian 16 package^61^. 4-Methylbenzenesulfinate and methanaminium were used as models of SO_3_^−^ and NH_3_^+^ according to their actual structures, respectively. The equilibrium geometries of the isolated ions and their complexes were optimized using the B3LYP functional^62,63^ with D3(BJ) empirical dispersion correction by Grimme et al.^64^ and ma-def2-TZVP basis set^65,66^. After the structure optimization, the energies of the optimized structures were calculated at B3LYP-D3(BJ)/ma-def2-TZVP level. All calculations were performed in the polarizable continuum model (PCM) with both dimethyl sulfoxide and vacuum as the medium^67^. The binding energies (*E*_b_) between different ions were calculated as follows:

$$E_{{{\mathrm{b}}}} = E_{{{{\mathrm{AB}}}}} - E_{{{\mathrm{A}}}}-E_{{{\mathrm{B}}}}$$

where *E*_AB_ represents the total energy of the ions A and B, *E*_A_ and *E*_B_ represent the energy of A and B, respectively.

## Supplementary information

supplementary information

## Data Availability

The data that support the plots within this paper and the other findings of this study are available from the corresponding authors upon reasonable request.
