# Supplementary material for: Rational adjustment to interfacial interaction with carbonized polymer dots enabling efficient large-area perovskite light-emitting diodes
Source: Light Sci Appl. 2023 May 15;12:119. doi: 10.1038/s41377-023-01150-1 (PMC10185670; doi:10.1038/s41377-023-01150-1)
Supplement: Supplementary file 1 — supplementary information [file 41377_2023_1150_MOESM1_ESM.docx]

**Supporting Information for**

**Rational Adjustment to Interfacial Interaction with Carbonized Polymer Dots Enabling Efficient Large-Area Perovskite Light-Emitting Diodes**

Fan Yang^1^, Qingsen Zeng^1, 4^, Wei Dong^2^, Chunyuan Kang^1^, Zexing Qu^3^, Yue Zhao^1^, Haotong Wei^1^, Weitao Zheng^2^, Xiaoyu Zhang^2^* and Bai Yang^1^*

^1^State Key Laboratory of Supramolecular Structure and Materials, College of Chemistry, Jilin University, Changchun, 130012, P. R. China.

^2^Department of Materials Science, Key Laboratory of Mobile Materials MOE, State Key Laboratory of Automotive Simulation and Control, Jilin University, Changchun, 130012, China.

^3^Institute of Theoretical Chemistry and Laboratory of Theoretical & Computational Chemistry, Jilin University, Changchun 130023, P. R. China

^4^Department of Materials Science and Engineering, Seoul National University, 1 Gwanak-ro, Gwanak-gu, Seoul 08826, Republic of Korea

*Corresponding authors: Prof. Xiaoyu Zhang ([zhangxiaoyu@jlu.edu.cn](mailto:zhangxiaoyu@jlu.edu.cn)); Prof. Bai Yang ([byangchem@jlu.edu.cn](mailto:byangchem@jlu.edu.cn))

**Supplementary Figures**


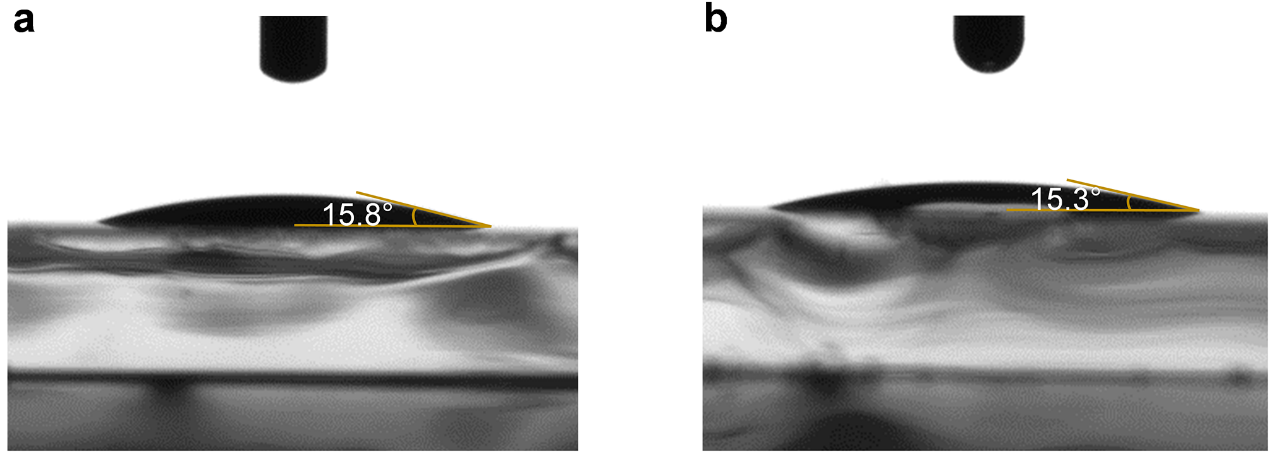


**Fig. S1: Perovskite precursor solution contact angles on different substrates at the fourth second. a** On pristine PEDOT:PSS substrate (p-HTL). **b** On CPDs-incorporated PEDOT:PSS substrate (CPD-HTL). Approximately 4 seconds pass between the first droplet contact and the start of spin coating.


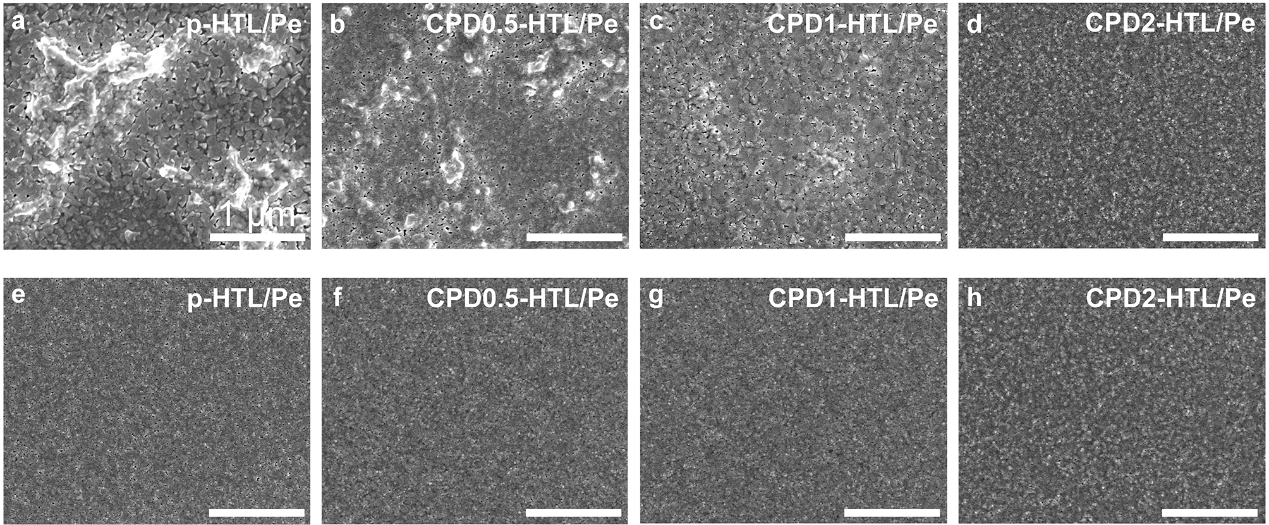


**Fig. S2:** **characterization of quasi-2D perovskite film morphology.** **a**-**c** Scanning electron microscopy (SEM) images of perovskite films on (**a**) p-HTL, (**b**) CPD0.5-HTL, and (**c**) CPD1-HTL in the ring region. **d** The SEM image of perovskite films on CPD2-HTL in the edge region. **e**-**g** SEM images of perovskite films on (**e**) p-HTL, (**f**) CPD0.5-HTL, and (**g**) CPD1-HTL in the non-ring region. **h** The SEM image of perovskite films on CPD2-HTL in the central region. Due to its flatness, the ring region of the CPD2-HTL/Pe is not visible, so SEM images are taken from the edge and central regions, in which both samples exhibit a dense and flat morphology.


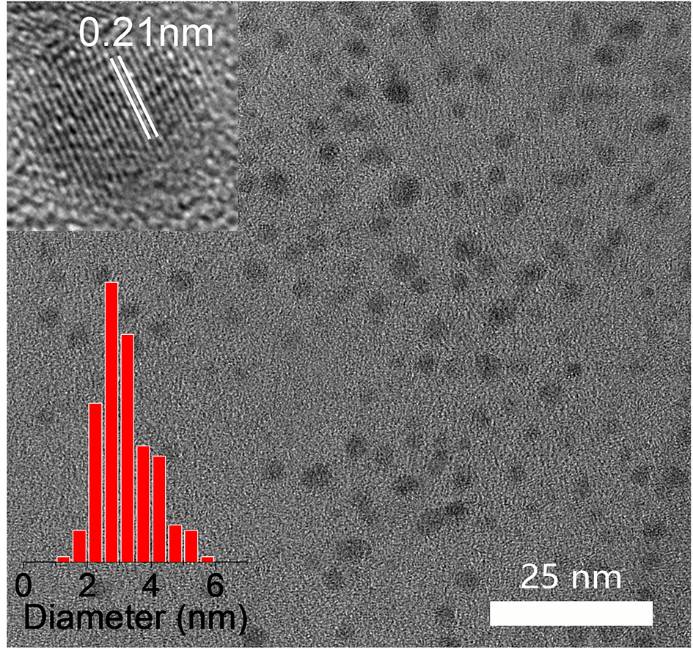


**Fig. S3: A typical TEM image, HRTEM image, and size distribution histogram for PA-EDA CPDs.** An average particle size of 3.17 nm was determined based on 150 particles. It is feasible to dope PA-EDA CPDs in PEDOT:PSS given their small size and high water dispersibility.


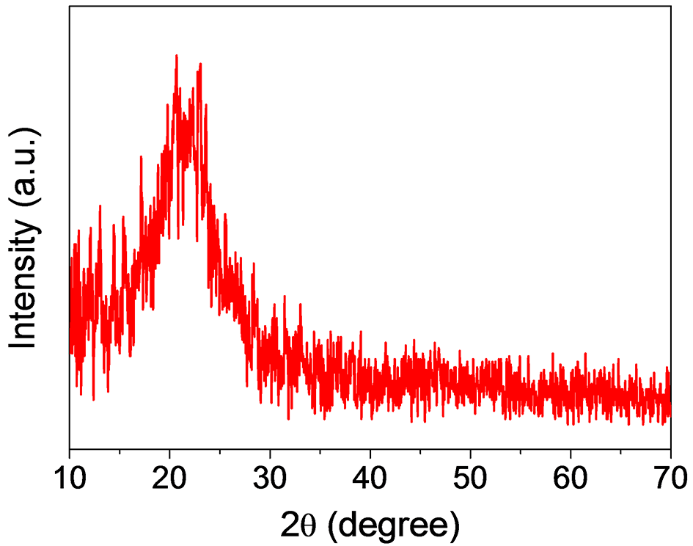


**Figure S4: The XRD pattern for PA-EDA CPDs.** CPD power shows only one broad peak at around 21.6°.


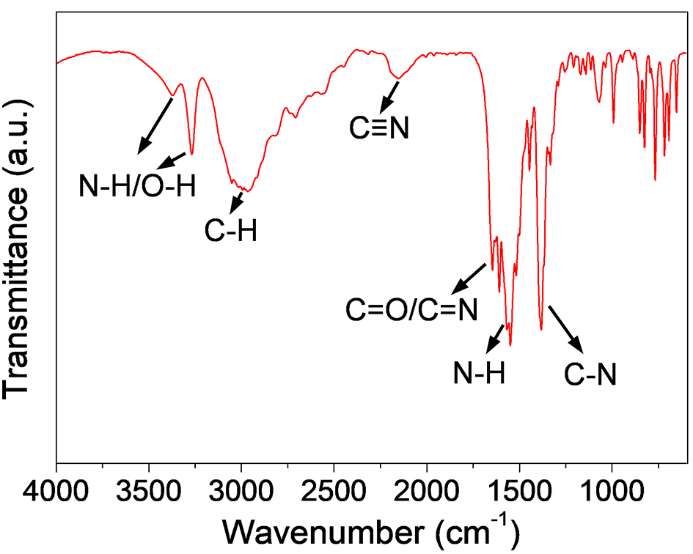


**Fig. S5: FTIR spectrum of the PA-EDA CPDs.** The band at 2152 cm^-1^ stems from C≡N and double bond accumulation region. The peak at 2966 cm^-1^ represents the C-H stretching vibration. The characteristic absorption bands for N-H (3373 and 1549 cm^-1^), C=O/C=N (1645 cm^-1^), and C-N (1382 cm^-1^) indicate the presence of amino groups, amide bonds, and carboxyl.


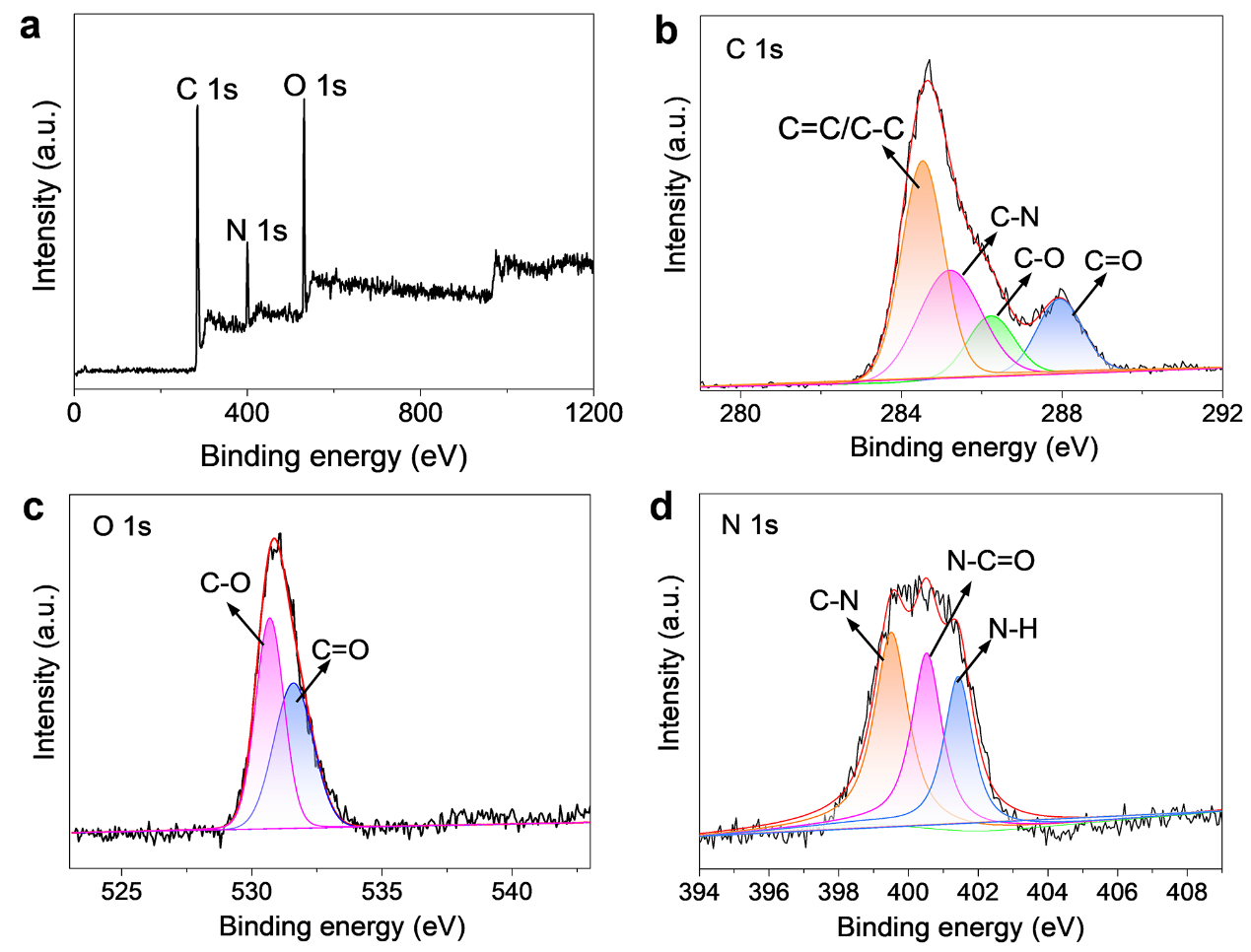


**Fig. S6: An XPS analysis of PA-EDA CPDs. a** XPS survey scan, **b** C 1*s*, **c** O 1*s*, **d** N 1*s* high resolution spectra of PA-EDA CPDs.

**a** The XPS survey scan reveals that PA-EDA CPDs are mainly composed of C (67.98%, atomic percentage), N (13.24%, atomic percentage), and O (18.79%, atomic percentage). N-containing groups are abundant in PA-EDA CPDs due to their high N content. **b** There are three different types of carbon atoms based on C 1*s* core level spectrum: graphitic or aliphatic carbon atoms (C=C/C−C, 284.8 eV), oxygenated carbon atoms (C=O/C−O, 288.2 eV, and 286.5 eV), and nitrogen-bearing carbon atoms (C−N, 285.5 eV). **c** In the O 1*s* band, there are peaks at 530.7 and 531.6 eV corresponding to C=O and C−O, respectively. **d** Three peaks at 399.7, 400.6, and 401.4 eV in the N 1*s* spectrum can be attributed to C-N, N-C=O, and N-H, respectively.


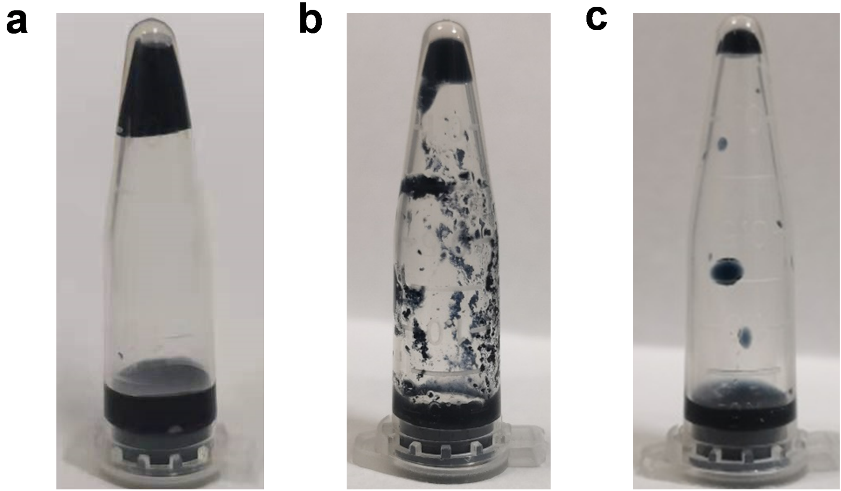


**Fig. S7: Evidence for CPDs-PEDOT:PSS interactions. a, b, c** Photographs of commercial PEDOT:PSS solutions mixed with (**a**) a small amount of PA-EDA CPDs, (**b**) large amounts of PA-EDA CPDs, and (**c**) excessive amino-free CPDs. The PEDOT: PSS blend is composed of insoluble PEDOT and hydrophilic PSS. Due to the strong electrostatic interaction between PA-EDA CPDs and PSS, PEDOT will be squeezed from the PSS polymer chains and precipitate in solution when excessive PA-EDA CPDs are added.


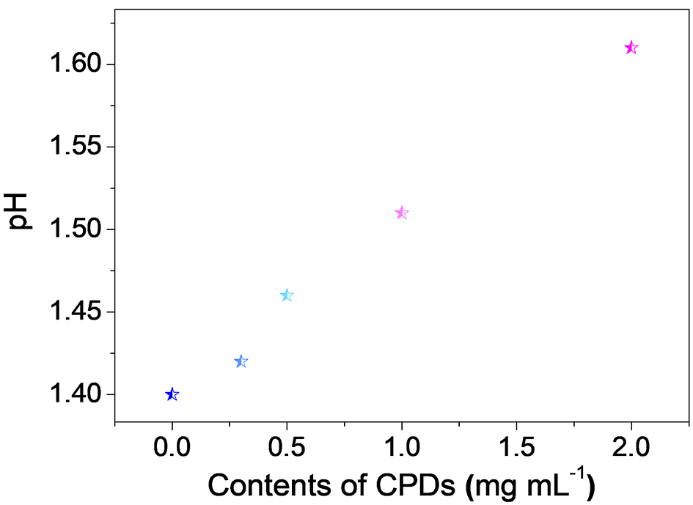


**Fig. S8: pH values of CPD-PEDOT:PSS mixtures.** A concentration increase of PA-EDA CPDs increases the pH value of the mixed solution of PEDOT:PSS and CPDs.


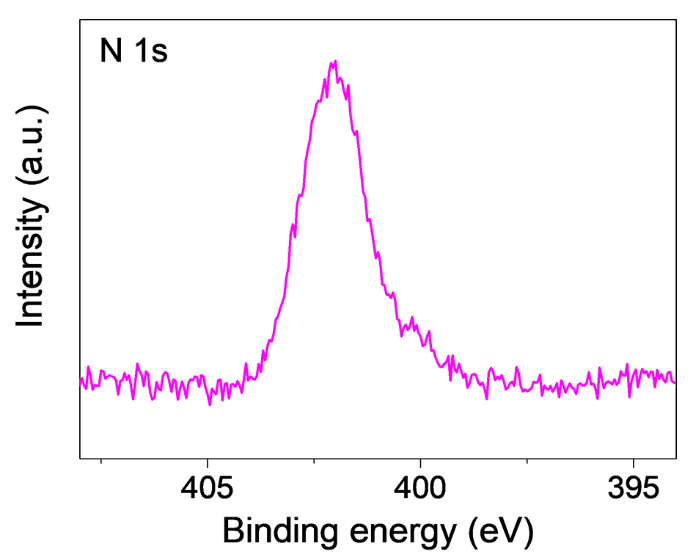


**Fig. S9: N 1*s* core level peak of a CPD-PEDOT:PSS film.** It appears that N atoms are concentrated on CPDs, whose peak position is 402 eV.


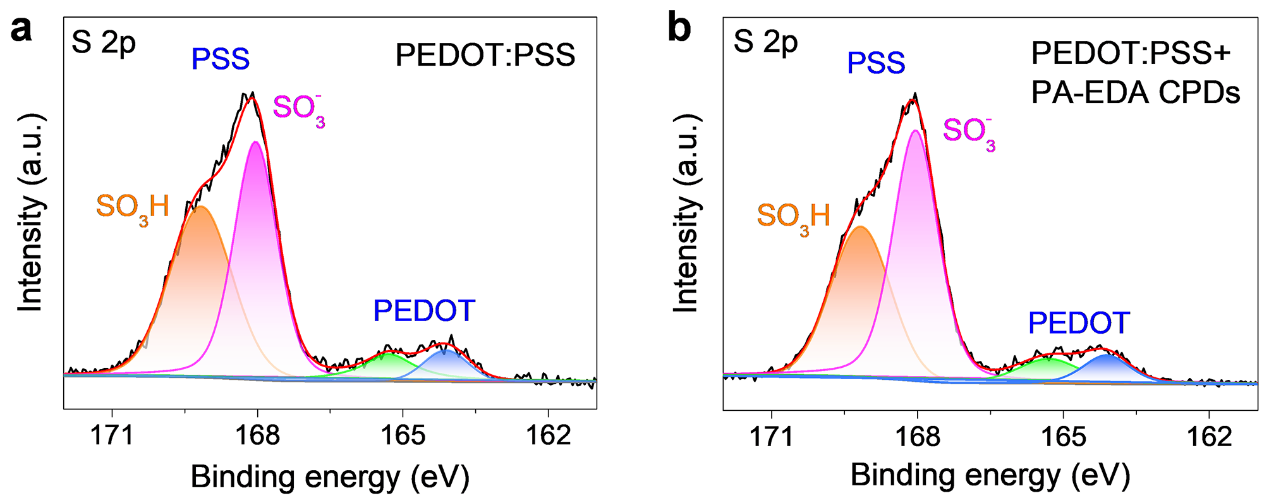


**Fig. S10: S 2*p* core level XPS spectra of different HTLs. a** Pristine PEDOT:PSS film. **b** CPD-PEDOT:PSS film. Both films show two characteristic S 2*p* peaks: one with higher binding energy of 166-171 eV, corresponding to sulfur atoms in PSS units, and the other with lower binding energy of 163-166 eV, representing sulfur atoms in PEDOT units. The spin-split peaks at 169.2 eV and 168.0 eV derive from the neutral sulfonic acid group (-SO_3_H) and the ionic sulfonate group (-SO_3_^-^).


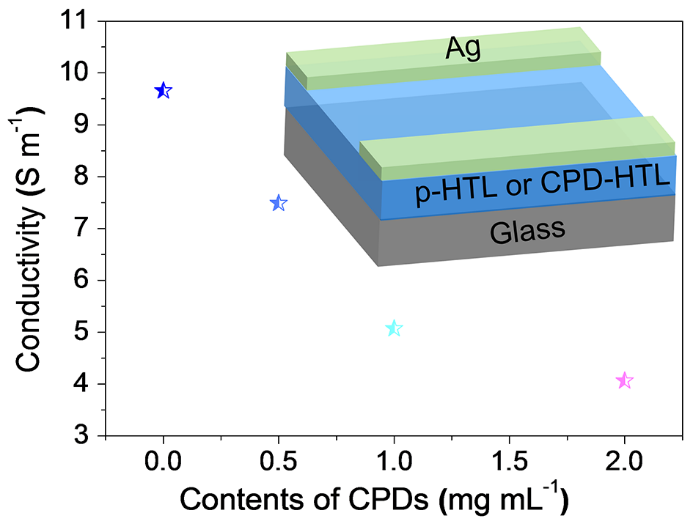


**Fig. S11: Electrical conductivity of PEDOT:PSS films containing different PA-EDA CPD contents.** Insert is the device configuration for measuring conductivity.

The conductivity formula is as follows:

*σ*=*L*/*SR*

where *σ* is the electrical conductivity, *L* is the length of film between two electrodes, *S* is the cross-sectional area of the film, and *R* is the slope of *V*-*I* curves.


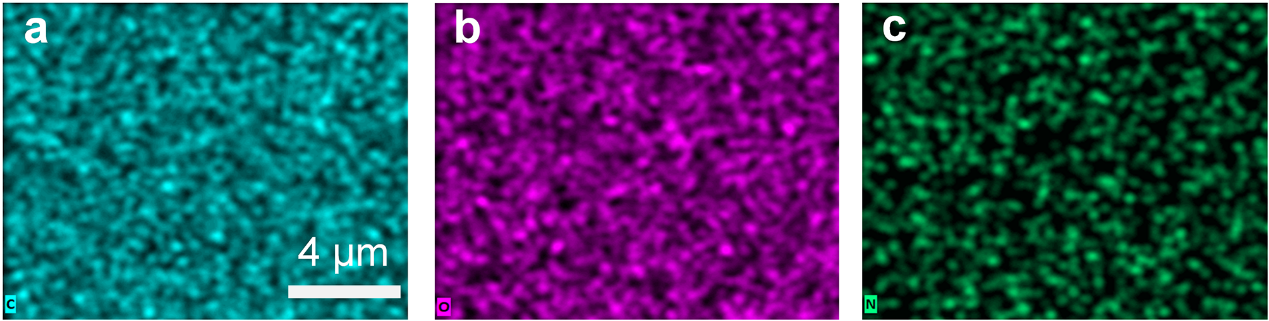


**Fig. S12: Distribution of elements in CPD-HTL. a, b, c** Energy dispersive spectroscopy (EDS) elemental mapping shows the distribution of (**a**) C, (**b**) O, (**c**) N elements. A homogeneous element distribution indicates uniform dispersion of PA-EDA CPDs in PEDOT:PSS films. All scale bars are 4 μm.


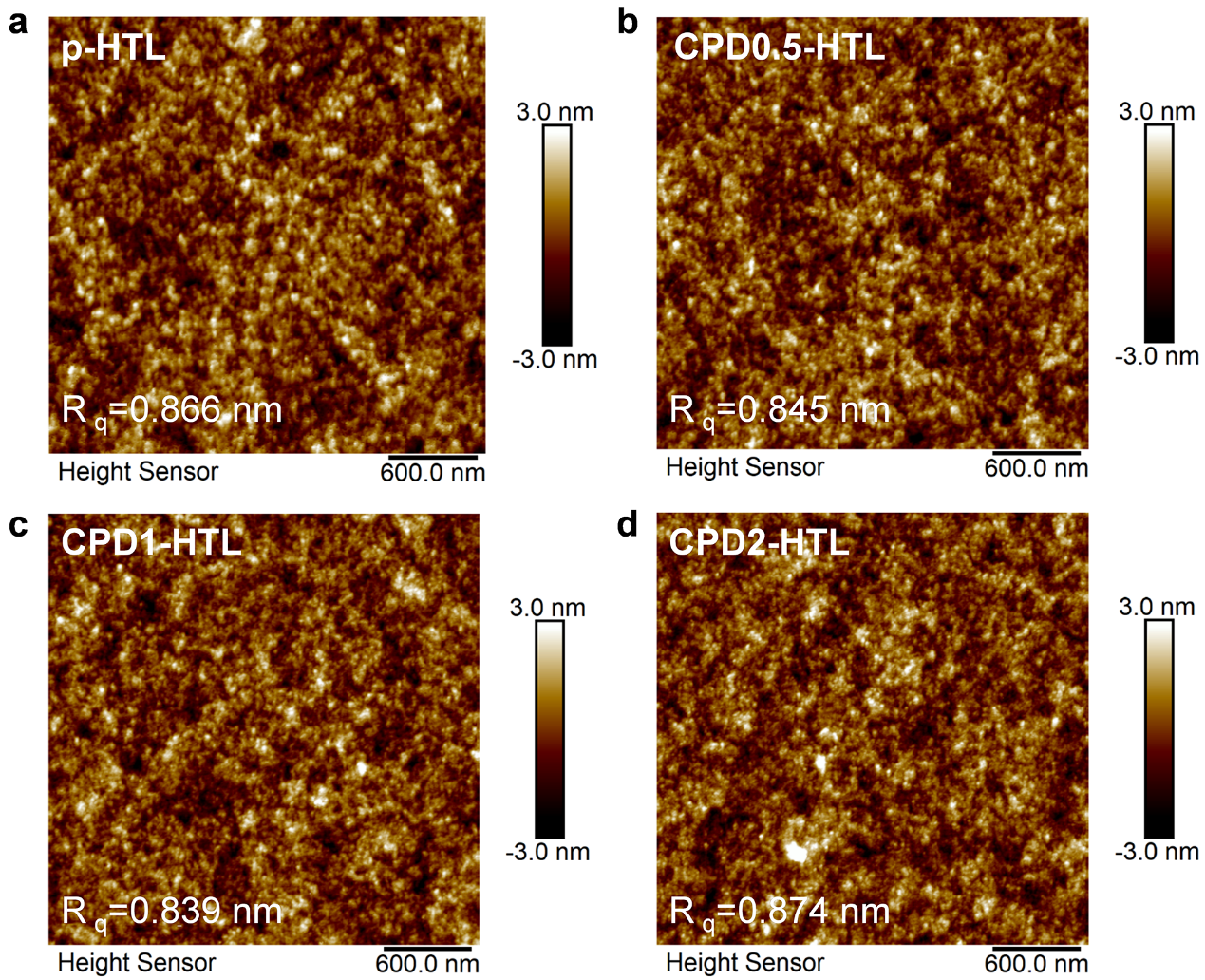


**Fig. S13: HTL morphology.** The following AFM images show HTL spin-coated using PEDOT:PSS solution with various PA-EDA CPD concentrations: **a** 0 mg mL^-1^, **b** 0.5 mg mL^-1^, **c** 1 mg mL^-1^, **d** 2 mg mL^-1^. All scale bars are 600 nm. It can be seen from AFM images that the addition of PA-EDA CPDs did not affect the surface roughness of HTL.


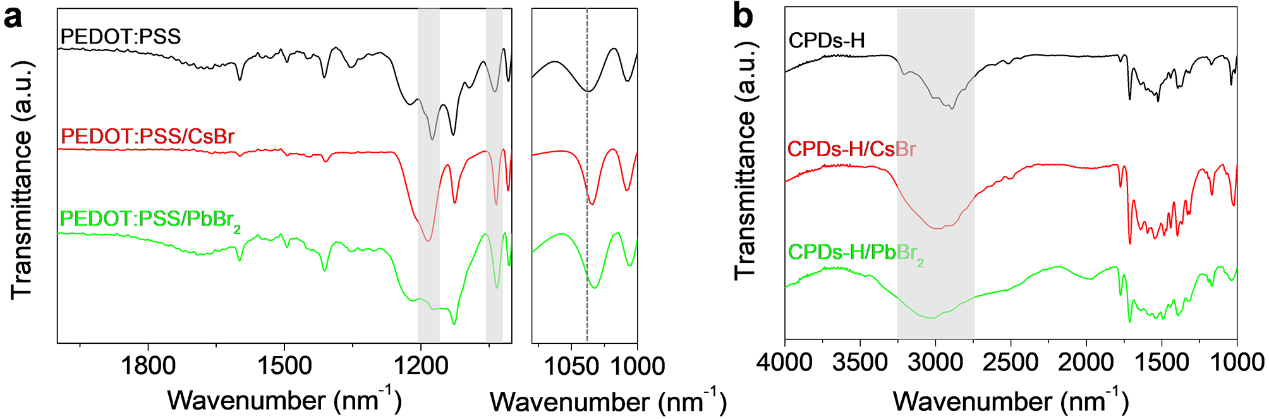


**Fig. S14: Characterization of interactions by FTIR spectroscopy. a** FTIR spectra of PEDOT:PSS and their mixtures with CsBr or PbBr_2_. **b** FTIR spectra of protonated CPDs (CPDs-H) and their mixtures with CsBr or PbBr_2_.

**
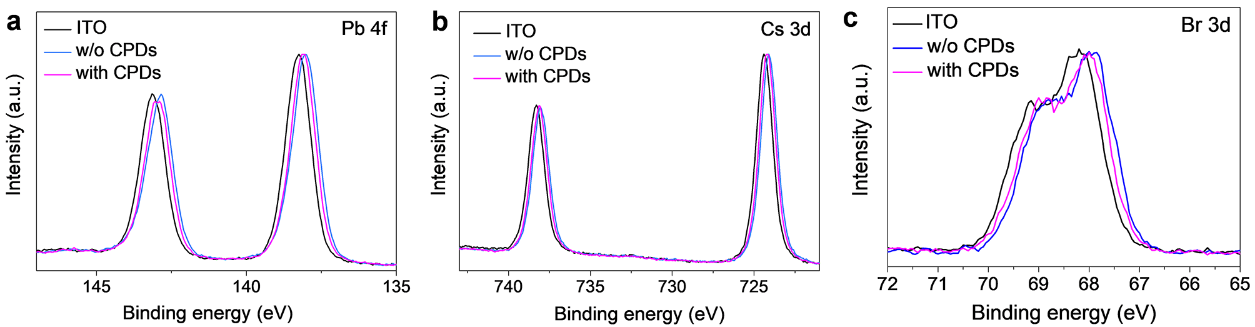
**

**Fig. S15: Characterization of interactions by XPS measurement. a, b, c** XPS spectra of (**a**) Pb 4*f*, (**b**) Cs 3*d*, and (**c**) Br 3*d* peaks of perovskite thin films prepared on bare ITO glass, PEDOT:PSS substrate, and CPD-PEDOT:PSS substrate. The XPS signals of Pb 4*f* and Cs 3*d* show a significantly reduced binding energy for perovskites on PEDOT:PSS than on ITO glass due to the electron donation from S=O. However, these peak shifts are less significant after the incorporation of CPDs. The Br 3*d* for perovskites on CPD-PEDOT:PSS shows a higher binding energy than on PEDOT:PSS as well, which should be attributed to the electrostatic interaction between -NH_3_^+^ and Br^-^. It is worth noting that the Br 3*d* peak of perovskites on ITO glass shifts a lot towards higher binding energy than on PEDOT:PSS or on CPD-PEDOT:PSS, which is due to the abundant In^3+^ and Sn^2+^ dangling bonds on ITO that interact with Br^-^ and reduce the electron cloud density of Br^-^.


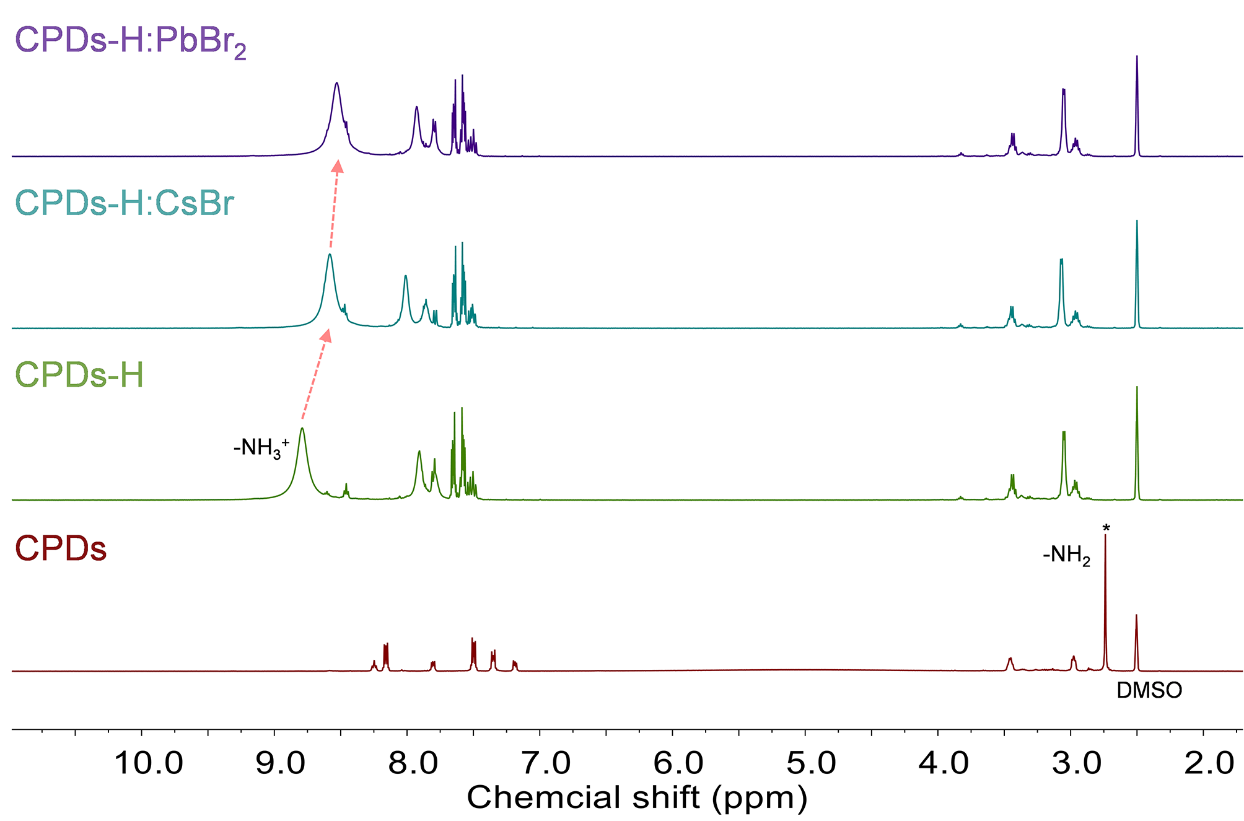


**Fig. S16: Characterization of interactions by ^1^H NMR.** ^1^H NMR spectra of CPDs, CPDs-H, CPDs-H mixed with CsBr, and CPDs-H mixed with PbBr_2_ in DMSO-d6.


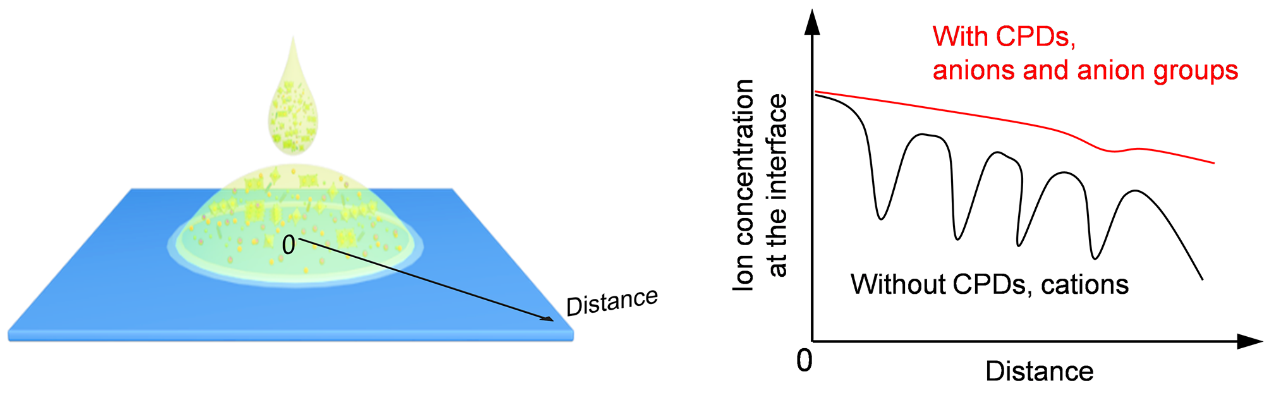


**Fig. S17: Schematic diagram of ion concentration versus distance at different interfaces.** Since the perovskite precursor solution is well infused into the HTL substrate, the droplets will spread outward quickly. Grain-growth substrates adsorb specific ions through interactions, causing changes in ion concentrations at interfaces. Without CPDs, pristine substrates have strong interactions with cations, resulting in a dramatic decrease in cation concentrations at the interface. With CPDs, the interaction turns to anions and anion groups, exhibiting a slight interference due to the higher concentration of anions and anion groups.

**
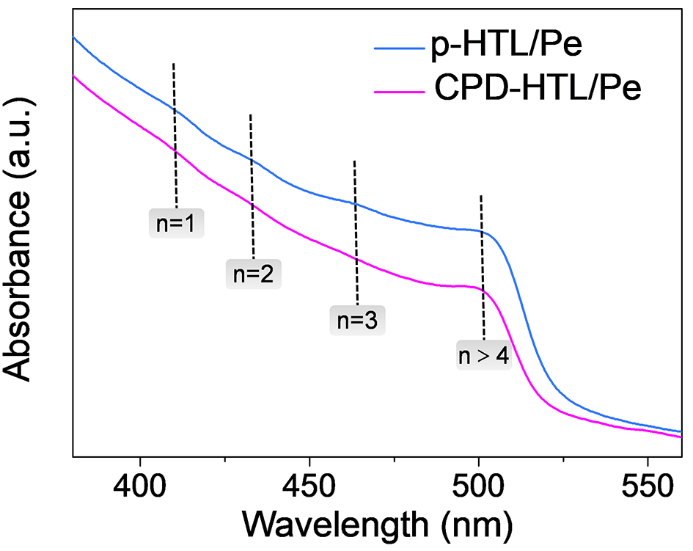
**

**Fig. S18: Absorption spectra of quasi-2D perovskite films.** The blue line represents the quasi-2D perovskites built on p-HTL. The pink line represents the quasi-2D perovskites prepared on CPD-HTL.

**
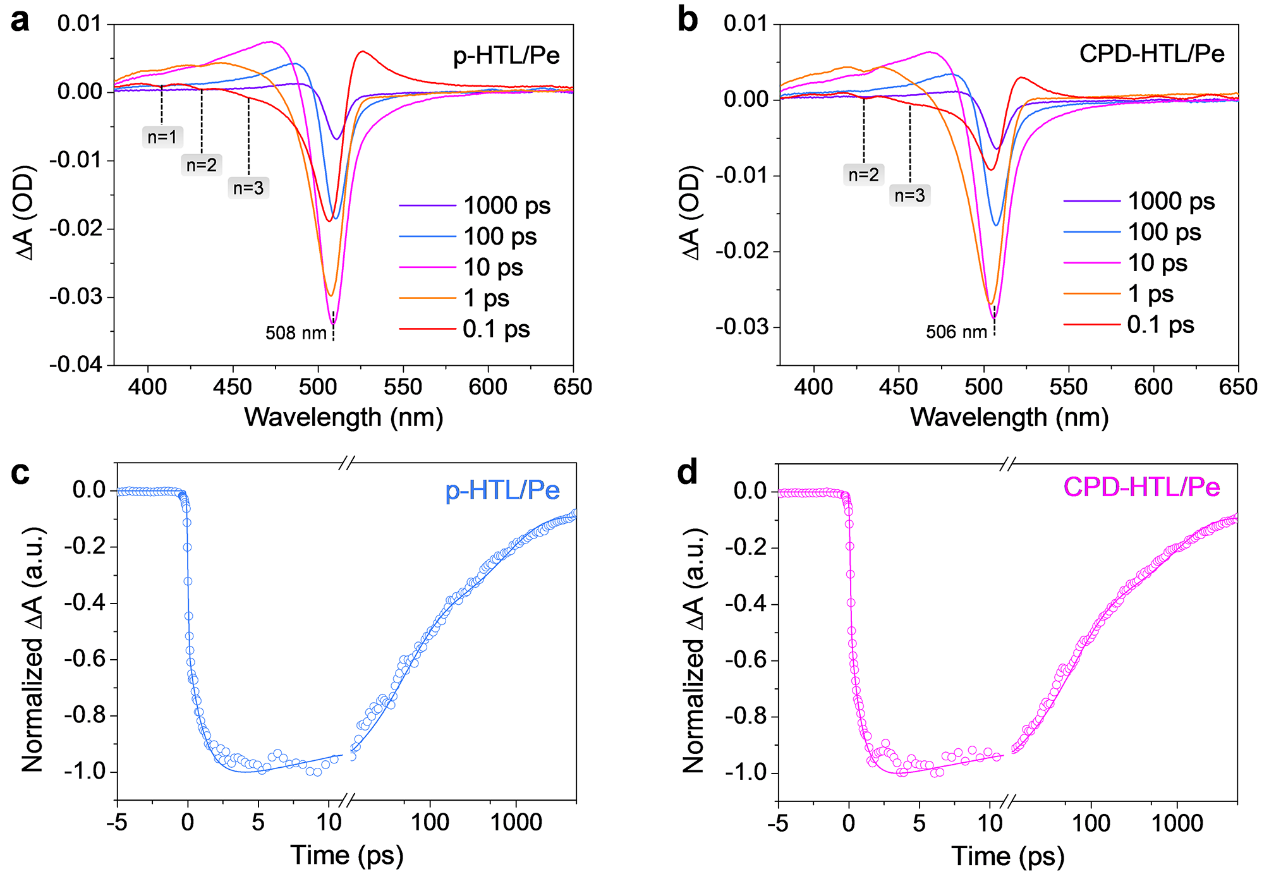
**

**Fig. S19:** **TA spectra of quasi-2D perovskite films. a**, **b** TA spectra at selected timescales for (**a**) p-HTL/Pe and (**b**) CPD-HTL/Pe films. **c**, **d** TA kinetics probed at *n*=∞ phase for (**c**) p-HTL/Pe and (**d**) CPD-HTL/Pe films.


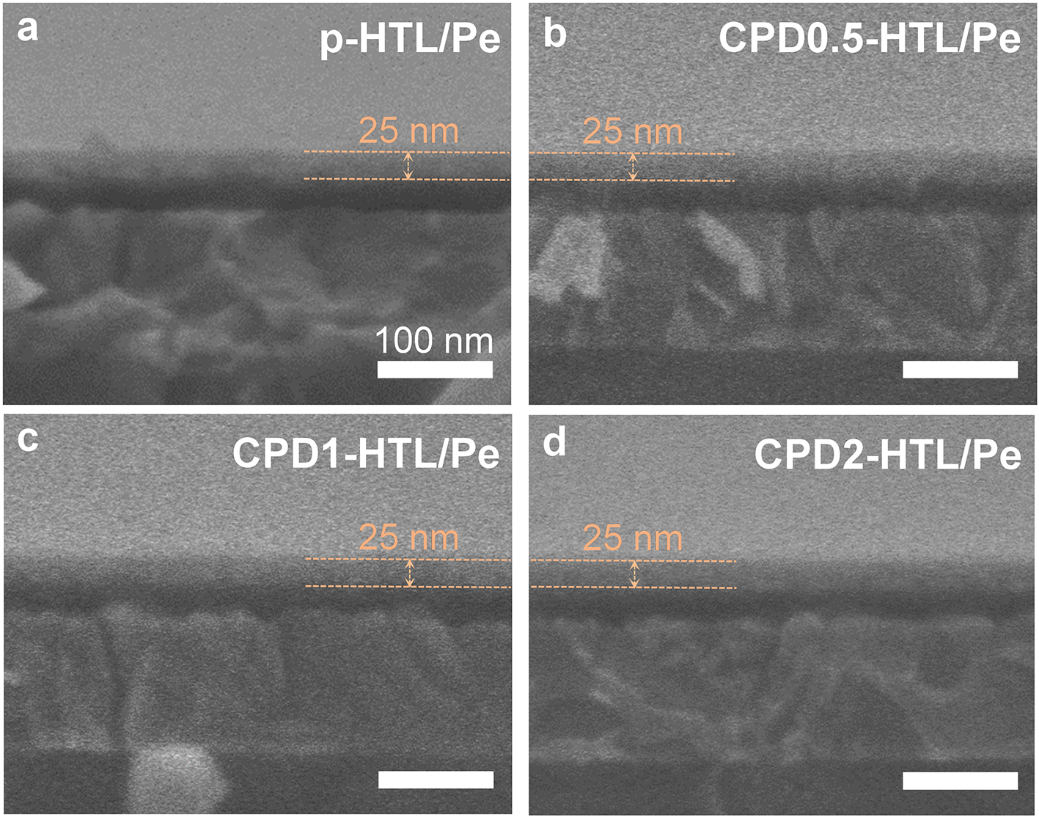


**Fig. S20: Characterization of perovskite film thickness.** Cross-section SEM images of quasi-2D perovskite films prepared on (a) p-HTL, (b) CPD0.5-HTL, (c) CPD1-HTL, and (d) CPD2-HTL. All scale bars are 100 nm.


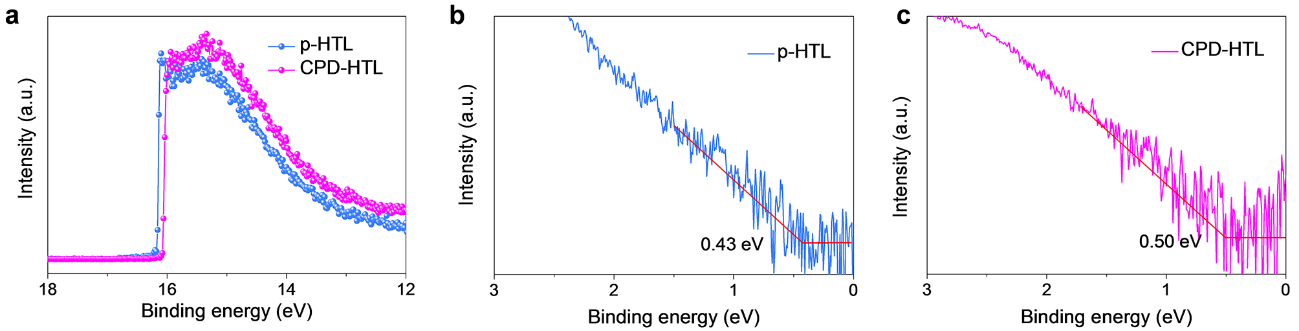


**Fig. S21: UPS spectra for different HTLs.** The blue line represents the pristine HTL. The pink line represents the CPD-HTL. For p-HTL and CPD-HTL, the secondary electron cutoff energy boundary (Ecutoff) and valence band maximum (VBM) onsets (*E*_onset_) are 16.17, 16.07, 0.43, and 0.50 eV, respectively. The corresponding VBM values are calculated as -5.48 and -5.65 eV based on the equation of *E*_VBM_= *E*_cutoff_ - *hν* - *E*_onset_.

**
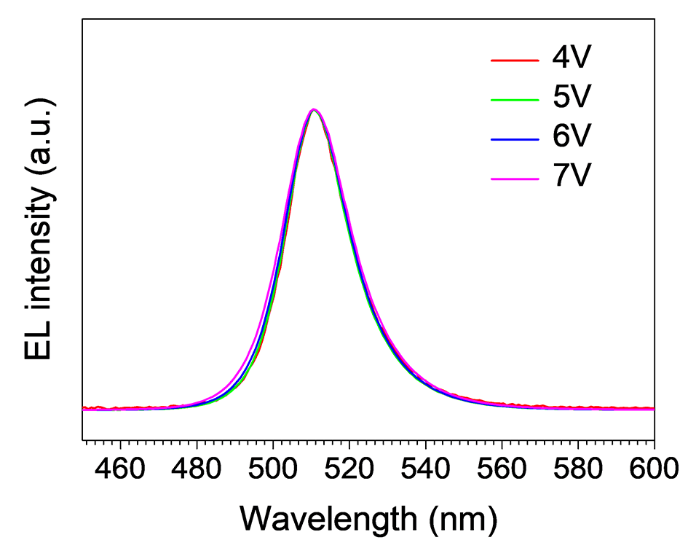
**

**Fig. S22: EL spectra of PeLEDs.** Normalized EL spectra at selected applied bias voltages of the PeLEDs with CPDs.

**
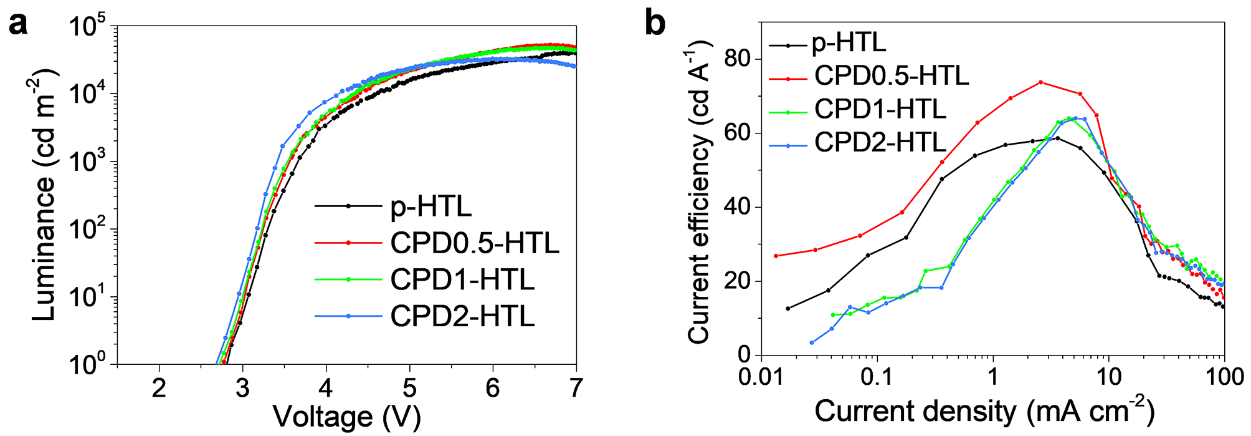
**

**Fig. S23: Characterizations of PeLEDs with different CPD concentrations. a** Luminance versus voltage. **b** Current efficiency versus current density.


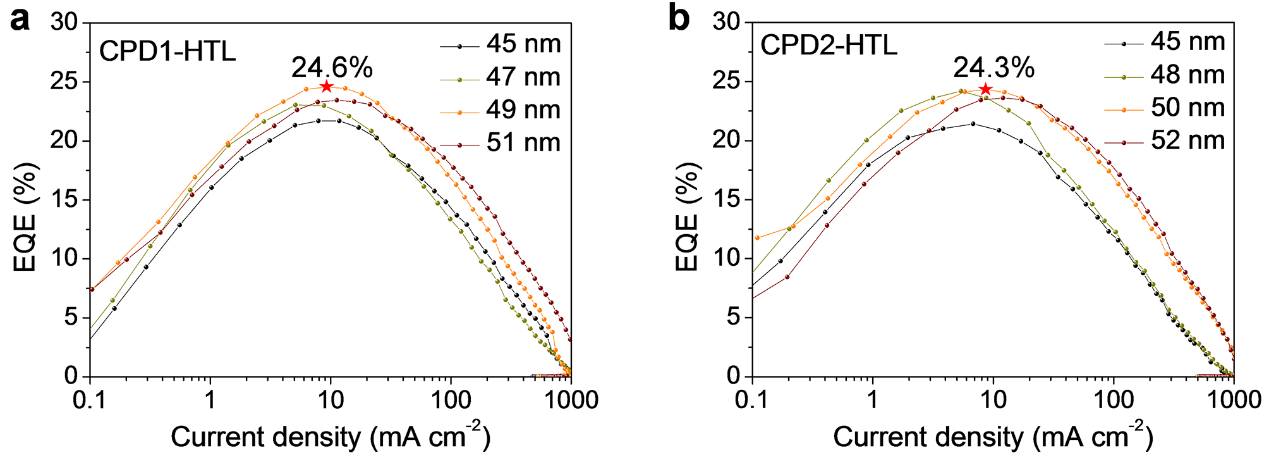


**Fig. S24: Performance optimization of devices with CPD1-HTL and CPD2HTL.** EQE versus current density at different thicknesses of TPBi for LEDs using **a** CPD1-HTL and **b** CPD2-HTL.


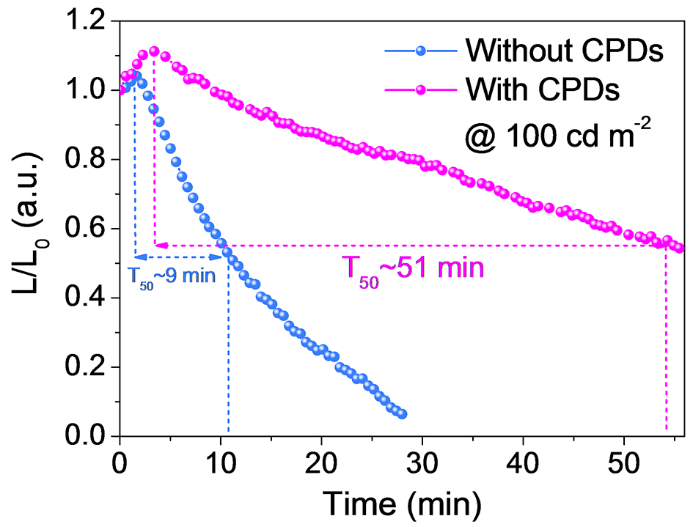


**Fig. S25:** **Device half-life measurements.** The operational stability of devices without and with CPDs (CPD1-HTL) was measured at the same initial luminance of 100 cd m^-2^ under a constant voltage.


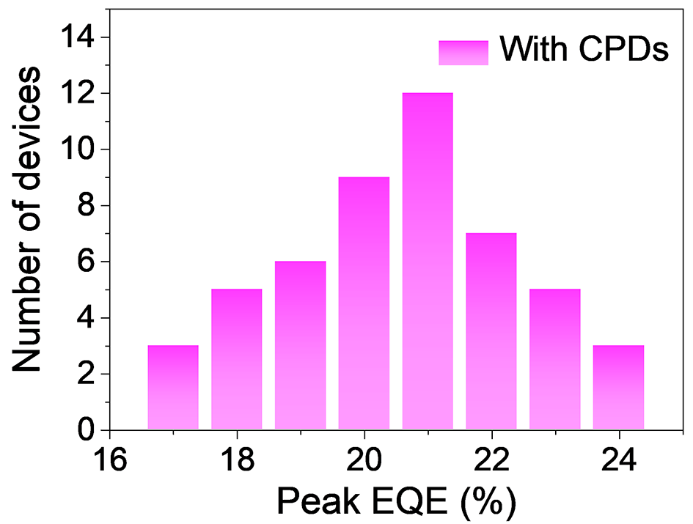


**Fig. S26:** **EQE histogram of PeLEDs with CPD1-HTL**. An average EQE of ~21% from 50 devices was demonstrated.


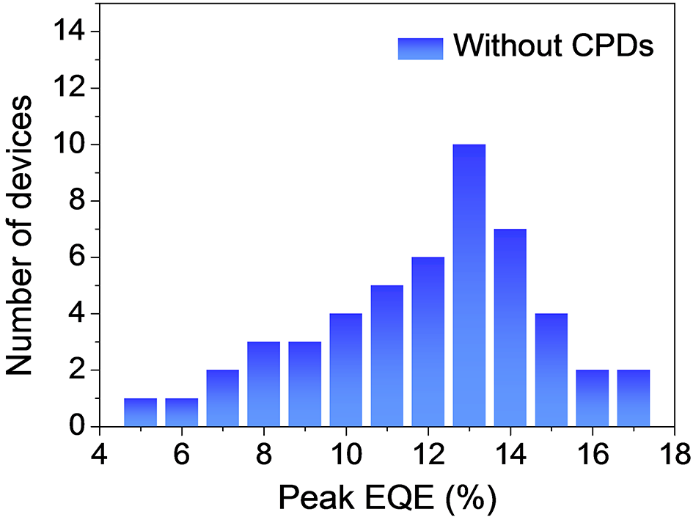


**Fig. S27: EQE histogram of PeLEDs without CPDs**.

**Supplementary Tables**

**Table S1:** A comparison between the peak areas of the splitting peaks, -SO_3_H (*A*$\text{-SO}_{\text{3}}\text{H}$) and -$\text{SO}_{\text{3}}^{\text{-}}$ (*A*$\text{-SO}_{\text{3}}^{\text{-}}$), and the area ratio (Fig. S10).

|  | *A*$\text{-SO}_{\text{3}}\text{H}$ | *A*$\text{-SO}_{\text{3}}^{\text{-}}$ | *A*$\text{-SO}_{\text{3}}\text{H}$/*A*$\text{-SO}_{\text{3}}^{\text{-}}$ |
| --- | --- | --- | --- |
| p-HTL | 4267.9 | 4476.7 | 0.953 |
| CPD-HTL | 5105.1 | 7345.7 | 0.695 |

**Table S2:** Fitting results of TA kinetics for perovskite films.

|  | *A*_1_ | *τ*_1_ (ps) | *A*_2_ | *τ*_2_ (ps) | *A*_3_ | *τ*_3_ (ps) |
| --- | --- | --- | --- | --- | --- | --- |
| p-HTL/Pe | 0.331 | 1.1 | -0.372 | 48.9 | -0.238 | 748 |
| CPD-HTL/Pe | 0.392 | 0.882 | -0.345 | 66.1 | -0.203 | 994 |

The kinetics of GSB peaks could be fitted by Surface Xplorer software using equation as follows:

Δ*A* (*𝜏*) = $e^{{-\left( \frac{\tau-\tau_{0}}{\tau_{p}} \right)}^{2}}$* (*A*_1_$e^{-\frac{\tau-\tau_{0}}{\tau_{1}}}$ + *A*_1_$e^{-\frac{\tau-\tau_{0}}{\tau_{2}}}$ + *A*_1_$e^{-\frac{\tau-\tau_{0}}{\tau_{3}}}$), *τ*_p_ = $\frac{IRF}{2ln2}$

where *IRF* is the width of instrument response function, *τ*_0_ is zero time, *τ*_1_, *τ*_2_, and *τ*_3_ are lifetimes, *A*_1_, *A*_2_, and *A*_3_ are amplitudes.
